# Collagen XIX is required for pheromone recognition and glutamatergic synapse formation in mouse accessory olfactory bulb

**DOI:** 10.3389/fncel.2023.1157577

**Published:** 2023-04-05

**Authors:** Chase Amos, Michael A. Fox, Jianmin Su

**Affiliations:** ^1^Center for Neurobiology Research, Fralin Biomedical Research Institute at Virginia Tech Carilion (VTC), Roanoke, VA, United States; ^2^School of Neuroscience, Virginia Tech, Blacksburg, VA, United States; ^3^Department of Biological Sciences, Virginia Tech, Blacksburg, VA, United States; ^4^Department of Pediatrics, Virginia Tech Carilion School of Medicine, Roanoke, VA, United States

**Keywords:** accessory olfactory bulb, Collagen XIX, glutamatergic synapse, synaptogenesis, pheromone recognition, development

## Abstract

In mammals, the accessory olfactory bulb (AOB) receives input from vomeronasal sensory neurons (VSN) which detect pheromones, chemical cues released by animals to regulate the physiology or behaviors of other animals of the same species. Cytoarchitecturally, cells within the AOB are segregated into a glomerular layer (GL), mitral cell layer (MCL), and granule cell layer (GCL). While the cells and circuitry of these layers has been well studied, the molecular mechanism underlying the assembly of such circuitry in the mouse AOB remains unclear. With the goal of identifying synaptogenic mechanisms in AOB, our attention was drawn to Collagen XIX, a non-fibrillar collagen generated by neurons in the mammalian telencephalon that has previously been shown to regulate the assembly of synapses. Here, we used both a targeted mouse mutant that lacks Collagen XIX globally and a conditional allele allowing for cell-specific deletion of this collagen to test if the loss of Collagen XIX causes impaired synaptogenesis in the mouse AOB. These analyses not only revealed defects in excitatory synapse distribution in these Collagen XIX-deficient mutants, but also showed that these mutant mice exhibit altered behavioral responses to pheromones. Although this collagen has been demonstrated to play synaptogenic roles in the telencephalon, those roles are at perisomatic inhibitory synapses, results here are the first to demonstrate the function of this unconventional collagen in glutamatergic synapse formation.

## Introduction

In mammals, the olfactory system plays an essential function in detecting odors and inducing responses to social behaviors, attraction, aversion, and fear. Rodents possess a number of odor sensing subsystems, including the main olfactory system (MOS) which is capable of detecting a broad range of odorants, the accessory olfactory system (AOS) which detects pheromone, septal organ and Grueneberg ganglion systems which have only recently been discovered and remain poorly understood (Ma, 2007; Sakano, 2020; Mori and Sakano, 2021). As the second largest of olfactory subsystem, the AOS consists of the vomeronasal organ (VNO), the accessory olfactory bulb (AOB), and the vomeronasal amygdala, which projects to hypothalamic neuroendocrine centers. Together this chemosensory system processes signals of non-volatile odorants emitted by animals, such as urine and feces, as well as fluids emanating from skin (Ma, 2007; Mohrhardt et al., 2018). Based on this, it was realized that AOS mainly functions in detecting and responding to pheromones, chemical cues which regulate the physiology or behaviors of other members of the same species (Liberles, 2014). Therefore, the AOB plays an important function in guiding physiological and behavioral responses to social and reproductive interactions.

The connectivity and cytoarchitecture of AOB are well characterized (Holy, 2018; Mohrhardt et al., 2018). Specifically, the AOB receives input from vomeronasal sensory neurons (VSN) whose axons extend *via* the cribriform plate, transverse along the medial aspect of the olfactory bulb, and terminal in the glomerular layer (GL) of AOB (Meredith, 1991; Belluscio et al., 1999; Rodriguez et al., 1999; Holy, 2018; Mohrhardt et al., 2018). Contrary to the MOS, individual VSN axons can divide to terminate in multiple glomeruli in the AOB rather than targeting single glomerulus in the MOB (Larriva-Sahd, 2008; Mohrhardt et al., 2018).

**FIGURE 1:**
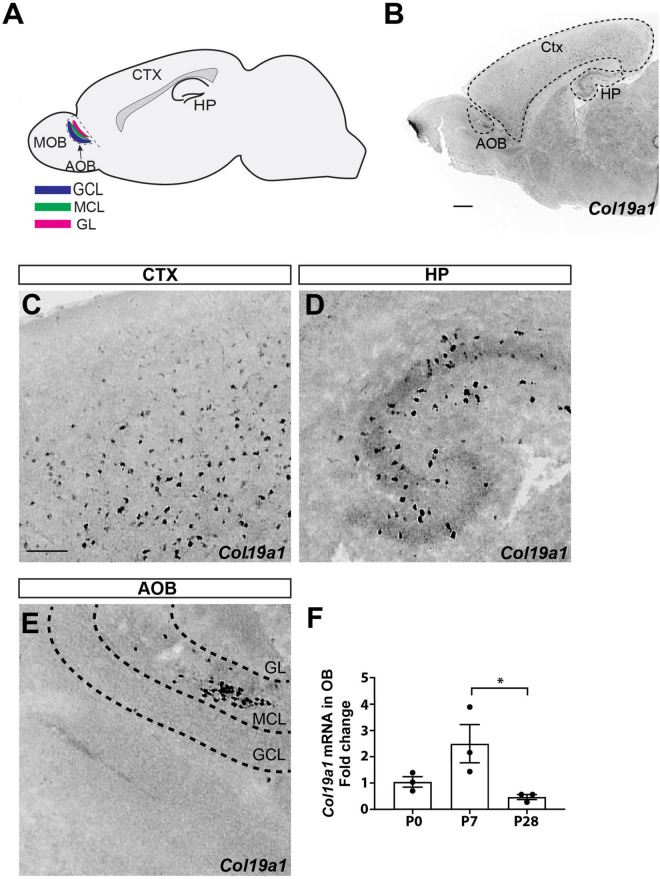
Spatial and temporal expression of *Col19a1*. **(A)** Schematic representation of the accessory olfactory bulb (AOB) in a sagittal view of mouse brain. Granule cell layer (GCL), mitral cell layer (MCL), and glomerular cell layer (GL) are each labeled. Cortex (Ctx) and hippocampus (HP) are also indicated. **(B)** *Col19a1* ISH performed on a sagittal wildtype mouse brain (P8). **(C–E)** Magnified views are shown from **(B)**, with expression of *Col19a1* in the Ctx **(C)**, HP **(D)**, and MCL of the AOB **(E)**. **(F)** *Col19a1* mRNA measured with qPCR of cDNAs generated from olfactory bulb RNA collected at birth (P0), P7, and P28. *Indicates *p* < 0.05 with multiple comparisons ANOVA/Tukey’s *post hoc*. Scalebars indicate 200 μm.

The AOB is situated at the posterior dorsal aspect of the rodent olfactory bulb. It is composed of a GL, mitral cell layer (MCL), granule cell layer (GCL), and narrow lateral olfactory tract (LOT) which is flanked by the MCL and GCL (Figure 1A; Yokosuka, 2012; Mohrhardt et al., 2018). The GL includes clustered glomeruli which are surrounded by periglomerular cells. The MCL contains AOB mitral cells (AMC), juxtaglomerular neurons (JGNs), and some external granule cells (eGCs). It is the dendrites of these AMCs that receive excitatory input from VSN axons in the AOB glomeruli (Brignall et al., 2018). The GCL is mainly occupied by axonless GABAergic internal granule cells (iGCs). The most prominent feature in this layer are the reciprocal dendrite to dendrite synapse between mitral and granule cell dendrites: mitral-to-granule cell synapses are glutamatergic, whereas granule-to-mitral cell synapses are GABAergic (Hayashi et al., 1993; Jia et al., 1999; Taniguchi and Kaba, 2001).

Although the cells and circuitry of mouse AOB has been well studied, the molecular mechanism underlying the assembly of such circuitry remains unclear. With the goal of identifying synaptogenic mechanisms in AOB, our attention was drawn to Collagen XIX, a neuronally produced non-fibrillar collagen that has previously been shown to regulate the assembly of synapses in the mouse telencephalon. Collagen XIX is necessary for inhibitory synapse formation in mouse hippocampus and neocortex. A short C-terminal fragment containing the non-collagenous domain 1 (NC1 domain) fragment of Collagen XIX, termed NC1[XIX] is sufficient to trigger inhibitory synapse formation in cultured cortical neurons (Sumiyoshi et al., 1997; Su et al., 2010, 2016, 2020). Our transcriptional analysis revealed that *Col19a1*, the gene that encodes Collagen XIX, is generated in the MCL of AOB. Here we tested whether the loss of Collagen XIX impaired synaptogenesis and the response to AOB-related pheromones using several genetic tools, including a targeted mouse mutant that lacks Collagen XIX globally and a conditional allele allowing for cell-specific deletion of this collagen (Sumiyoshi et al., 2004; Su et al., 2020). Our data show that both the global loss Collagen XIX mice and the cell-specific loss of Collagen XIX mice exhibit pheromones sensing and excitatory synapse formation defects.

## Materials and methods

### Animals

C57BL/6 (Charles River Laboratories) and previously generated Collagen XIX-null mice (Sumiyoshi et al., 2004; Su et al., 2010) were used in this study. Additionally, *Col19a1^fl/fl^*, *Nes-cre::Col19a1*^fl/fl^**, and *Sst-cre::Col19a1*^fl/fl^** mice were generated as previously described (Su et al., 2020) from The Jackson Laboratory *Nes-cre* (#003771), *Vglut2-cre* (#016963) and *Sst-cre* (#013044) mice. Mice were housed in a 12 h dark/light cycle and had *ad libitum* access to food and water. All experiments were performed in compliance with National Institutes of Health (NIH) guidelines and protocols, and were approved by the Virginia Polytechnic Institute and State University Institutional Animal Care and Use Committee (IACUC).

### Reagents

The following materials were obtained, from Roche (Basel, Switzerland): Fluorescein RNA Labeling Mix, digoxigenin (DIG) RNA Labeling Mix, blocking reagent, and Yeast RNA; from EMS (Hatfield, PA): Paraformaldehyde (PFA, EM-grade) and Tissue Freezing Medium; from Vector Laboratories (Newark, CA): VECTASHIELD; from Bio-Rad (Hercules, CA): Aurum Total RNA Fatty and Fibrous Tissue Kit; from Promega (Madison, WI): pGEM-T Easy Vector Systems; from PerkinElmer (Waltham, MA): tyramide signal amplification (TSA) systems. All other chemicals and reagents were obtained from Thermo Fisher Scientific or Sigma-Aldrich, and all DNA primers from Integrated DNA Technologies.

### *In situ* hybridization (ISH)

Riboprobes against *Col19a1*, *Gad1*, *Syt1*, *Syt2*, *Vglut1* and *Sst* mRNAs were generated as described previously (Su et al., 2010, 2016, 2020; Carrillo et al., 2023), where digoxigenin-labeled NTP (Roche, Basel, Switzerland) and the MAXIscript *In Vitro* Transcription Kit (Ambion) were used to synthesize riboprobes, which were hydrolyzed to 500 nt. Prior to addition of the riboprobes, tissue sections were prepared for ISH by fixation with 4% paraformaldehyde (PFA) for 10 min, incubation with proteinase K (1 ug/mL proteinase K, 50 mM 7.5 pH Tris, 5 mM EDTA) for 10 min, additional fixation with 4% PFA for 5 min, acetylation (1.33% triethanolamine, 20 mM HCl, 0.25% acetic anhydride) for 10 min, and permeabilization (1% Triton X-100) for 30 min, where each step listed was followed by DEPC-PBS washes. After fixation and permeabilization, sections were blocked with 0.3% H_2_O_2_ for 30 min, incubated with a hybridization buffer (1X prehybridization, 0.1 mg/mL yeast tRNA, 0.05 mg/mL heparin, 50% formamide) for 1 h, and incubated overnight with riboprobes at 65°C, concluded by a 65°C wash with 0.2x SSC. Horseradish peroxidase (POD)-conjugated anti-DIG (1:2,000, Roche, Basel, Switzerland) or anti-FL (1:2,000, Roche, Basel, Switzerland) antibodies were used with TSA systems (PerkinElmer, Waltham, MA) fluorescent staining. VectaShield-mounted sections were imaged with a Zeiss LSM 700 microscope (20x air Plan-Apochromat Objective, NA 0.8).

### Immunohistochemistry

To prepare slides for IHC, mice were first anesthetized with 12.5 ug/mL tribromoethanol (Avertin). Blood was cleared with transcardial perfusion of phosphate-buffered saline (PBS) and subsequently 4% PFA to preserve the tissues. The brain was removed, incubated with 4% PFA for 16 h at 4°C for further fixation, and cryopreserved in 30% sucrose for ≥ 48 h at 4°C. After embedding the fixed tissue in tissue freezing medium (EMS), a Leica CM1850 cryostat was used to prepare 20 μm cryosections. Air-dried sections were then blocked with 2.5% BSA, 5% NGS, and 0.1% Triton-X in PBS for 1 h. Tissue sections were stained with primary antibodies in blocking buffer for 16 h at 4°C: mouse anti-Syt2 (diluted 1:200, Zebrafish International Resource Center), rabbit anti-VGluT1 (diluted 1:500, Synaptic Systems #135303), mouse anti-GAD67 (diluted 1:100, Millipore #MAB5406), rabbit anti-VGLUT2 (diluted 1:500, Synaptic Systems #135402), rabbit anti-IBA1 (diluted 1:1,000, Wako #019-19741), NeuN (diluted 1:250, Millipore), GFAP (diluted 1:1,000, DakoCytomation #Z 0334). After three washes with PBS, sections were incubated with fluorescent secondary antibodies (1:1,000, Invitrogen Life Technologies, Waltham, MA) for 1 h at 22°C. Following three additional washes with PBS, nuclei were stained with DAPI (1:5,000 dilution in water), and sections were subsequently mounted with Vectashield (Vector Laboratories). The sections were imaged with a Zeiss LSM 700 confocal microscope or a Zeiss AxioImager A1 fluorescent microscope. A minimum of three animals for each group were prepared in all IHC experiments.

### Quantitative real-time PCR (qPCR)

After isolation of RNA with BioRad Total RNA Extraction from Fibrous and Fatty Tissue kit (BioRad), cDNAs were generated from 200 ng RNA with the Superscript II Reverse Transcription First Strand cDNA Synthesis kit (Invitrogen). 10 ng RNA was combined with primers [5′-ATTGGACATAAGGGCGACAA-3′ and 5′-AGTCTCCTTTGGCTCCTGGT-3′ for *Col19a1* (Su et al., 2016) or 5′-GGACCAGAGCGAAAGCATTTG-3′ and 5′- GCCAGTCGGCATCGTTTATG-3′ for 18S] and the iQ SYBRGreen Supermix (BioRad). Quantitative real-time PCR (qPCR) was performed on a Chromo 4 Four Color Real-Time system (BioRad) with 1 cycle of 95°C for 30 s and 40 cycles of amplification (95°C for 5 s, 60°C for 30 s, 55°C for 60 s, read plate) and a melting curve analysis. Relative quantities of RNA were determined using the ΔΔ-CT method (Livak and Schmittgen, 2001). At least three samples (each in triplicate) were examined for each age.

### Olfactory preference test

Previously reported olfactory preference tests (Kobayakawa et al., 2007; Zou et al., 2015) were modified for usage with a three-chamber box, where a middle chamber openly connects to two flanking chambers (Stoelting, Wood Dale, IL). Removable doors initially blocked access to the flanking chambers from the empty middle chamber. A 2 cm×2 cm filter paper was introduced to each of the two flanking chambers. Male mice were separated with female mice at least 3 weeks before experiments. A male test mouse (3–5 months old) was habituated in the closed middle chamber for 5 min. Mouse urine was collected in 1.5 ml tubes. 20 μL of previously collected mouse urine was then pipetted onto each of the two filter papers as a source of pheromones, with male urine on one filter paper and female urine on the other filter paper. The testing period lasted for 3 min total, beginning with the simultaneous removal of the two doors blocking the flanking chambers. During the 3 min test period, the time of the testing mouse’s visit to each flanking chamber (male urine chamber, female urine chamber) was recorded to quantify olfactory preference of male versus female pheromones. A minimum of *n* = 9 mice was included in each group tested.

### Quantification of immunoreactivity

The intensity of presynaptic terminals in sections of accessary olfactory bulbs was measures by ImageJ. At least 3 mice were analyzed per genotype, and the mean values were compared between groups. Student’s *t*-test were used to determine the significance between control and mutant groups.

### Statistical analysis

Results are presented as bars indicating mean ± standard error of mean (SEM), with the datapoint of each mouse shown, unless otherwise indicated. Statistical analysis and preparation of graphs was carried out with GraphPad Prism 7. Comparisons of expression between different groups in Figure 1F was determined by performing a two-way analysis of variance (ANOVA test) followed by a Tukey *post-hoc* test. All other statistical analyses presented were performed by either a paired or unpaired Student’s *t*-test as indicated. No outlier tests or assessments of normality were conducted.

## Results

### *Col19a1* is specifically expressed in MCL of the AOB

In our previous studies on *Col19a1* expression in developing mouse neocortex we observed cellular expression of this ECM molecule in the olfactory bulb (Su et al., 2016; Figure 1B). To determine which type of cell generates *Col19a1* and in what region of the olfactory bulb, we performed *in situ* hybridization (ISH) using *Col19a1* riboprobes on sagittal section of mouse brains. As we reported before, robust *Col19a1* expression cells was observed throughout the cerebral cortex and hippocampus (Su et al., 2010, 2016; Figures 1B–D). In contrast to the dispersed cellular expression in telencephalon, we found that *Col19a1* expression in the olfactory bulb (OB) was both regionally and lamina specific: *Col19a1*-expressing cells were specifically distributed in the MCL of the AOB (Figures 1B, E) and anterior olfactory nucleus (Figure 1B and Supplementary Figure 1). We further examined the developmental regulation of *Col19a1* mRNA in mouse OB by qPCR. Our results showed that *Col19a1* expression was developmentally regulated. *Col19a1* expression peaked at 7 days postnatal, after which its expression was significantly decreased (Figure 1F).

### *Col19a1* is expressed by a distinct subset of inhibitory neurons in the AOB and CTX

**FIGURE 2:**
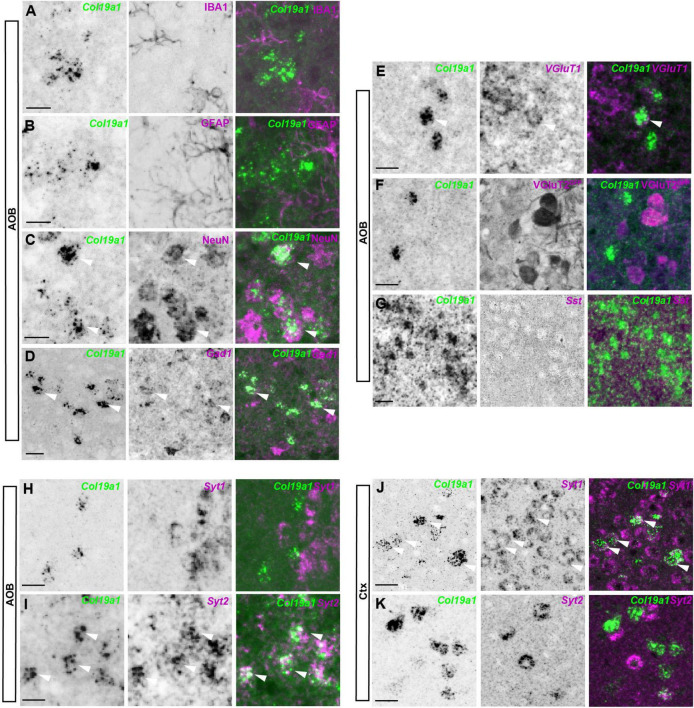
*Col19a1* is expressed by distinct subsets of inhibitory neurons in the accessory olfactory bulb (AOB) and cortex. **(A–G)** ISH with *Col19a1* anti-sense riboprobes was used to determine cell type expression in the AOB, Co-staining was performed with IBA1 (microglia) **(A)**, GFAP (astrocytes) **(B)**, NeuN (neurons) **(C)** and GFP (VGlut2-GFP, Excitatory neurons) **(F)** antibodies. Double *in situ* hybridization was performed with riboprobes against *Gad1* (inhibitory neurons) **(D)**, *Vglut1* (excitatory neurons) **(E)** or *Sst* (interneurons) **(G)**, where arrow heads indicate the co-expression of *Col19a1* by NeuN^+^, *Gad1*^+^, and *Vglut1*^+^cells. **(H–K)** Comparison of *Syt1* and *Syt2* co-expression by *Col19a1*^+^ Cells in the AOB **(H,I)** and Ctx **(J,K)**. Arrow heads indicate *Syt2*^+^ *Col19a1*^+^ cells in the AOB and examples of *Syt1*^+^ *Col19a1*^+^ and *Syt2*^+^ *Col19a1*^+^ in the Ctx. All images were acquired from sagittal tissue sections from P11-P14 wildtype mice. Scalebars indicate 20 μm.

Our previous studies showed that *Col19a1* mRNA was specifically generated by GABAergic neurons in the cortex and hippocampus (Su et al., 2010, 2016). We therefore hypothesized that *Col19a1* would similarly be expressed by GABAergic neurons in the OB. For this we performed *in situ* hybridization coupled to immunohistochemistry with antibodies against IBA1 (which labels microglia), GFAP (which labels astrocytes) and NeuN (which labels most neurons), respectively. We found that *Col19a1* was expressed by neurons but not by glia in mouse AOB (Figures 2A–C). We further performed double *in situ* hybridization for *Col19a1* mRNA and *Gad1* mRNA, which was generated by GABAergic neurons. In contrast to neocortex where *Col19a1* was exclusively expressed by GABAergic neurons only a fraction [68 ± 3% (*n* = 3)] of *Col19a1*^+^ cells co-expressed *Gad1* in the AOB (Figure 2D). More interestingly, When we tested *Vglut1* and *Vglut2*, which were generated by excitatory neurons, we found that a small part [23 ± 3% (*n* = 3)] of *Col19a1* was expressed by *Vglut1* positive neurons (Figure 2E), but there is no *Col19a1* was expressed by *Vglut2* positive neurons (Figure 2F). Furthermore, while *Col19a1* expression was largely restricted to Synaptotagmin 1 (*Syt1*) positive neurons in neocortex (Figures 2J, K), it was co-expressed by Synaptotagmin 2 (Syt2) positive neurons in the mouse OB (Su et al., 2010, 2016), (Figures 2H, I). Taken together, these data suggested *Col19a1* was generated by neurons in the developing AOB, but this appears to be a different type of neuron than in neocortex.

### Collagen XIX is required for pheromone recognition

**FIGURE 3:**
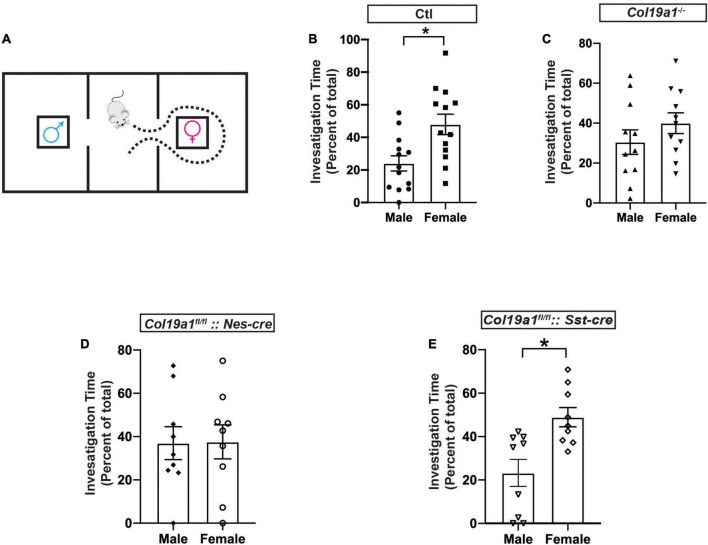
Olfactory function is disrupted in the absence of *Col19a1*. **(A)** Schematic for the male olfactory preference for female urine. A male mouse is placed in a three-room chamber, with female urine on filter paper in one chamber and male urine on filter paper in the other. **(B–E)** Comparison of male mouse preference for female urine over male urine between control (*n* = 13) **(B)**, *Col19a1*^– /–^ (*n* = 11) **(C)**, *Col19a1*^fl/fl^*: Nestin-cre* (*n* = 9) **(D)**, and *Col19a1*^fl/fl^*: Sst-cre* (*n* = 9) **(E)**. *Indicates *p* < 0.05 with a paired Student’s *t*-test.

Previous studies reported that the MCL of the AOB was critical for pheromone recognition preference (Pankevich et al., 2006), which led us to hypothesize that *Col19a1* may be involved in pheromone recognition. We measured male mice’s olfactory preference for female urine using a three-chamber box (Figure 3A). Male mice have a significant preference to investigate the chamber containing filter paper saturated with female urine, rather than a room containing filter paper soaked with male urine (Figure 3B). We first tested how mutant mice globally lacking Collagen XIX (*Col19a1*^–/–^) [Mice have no motor activity and anxiety-related phenotypes (Su et al., 2016, 2020)] performed in this pheromone preference assay. We found that *Col19a1*^–/–^ male mice spent similar time investigating the filter paper containing female or male urine, suggested that the loss of Collagen XIX led to impaired pheromone recognition (Figure 3C).

To assess whether brain-derived Collagen XIX was specifically necessary for pheromone preference, we crossed a conditional allele of *Col19a1* (*Col19a1^fl/fl^*) mice with *Nestin-cre* (*Nes-cre*) mice to conditionally delete Collagen XIX from mouse brain. We found that the deletion of brain-derived Collagen XIX caused mice to lose pheromone preference as well (Figure 3D).

Since we found that somatostatin (Sst) was not expressed in the AOB (Figure 2G), we then probed whether *Col19a1*^fl/fl^**:: *Sst*-cre mice would have changes in pheromone preference to rule out non-AOB contributions of Collagen XIX to this behavior. Similarly to wildtype mice, *Col19a1*^fl/fl^**:: *Sst*-cre male mice spent significant more time in a chamber with female urine (Figure 3E). We interpreted this to suggest that the loss of Collagen XIX from the AOB (and not neocortex or anterior olfactory nucleus) impaired pheromone recognition in mice.

### Loss of Collagen XIX disrupts excitatory synapse formation in AOB

**FIGURE 4:**
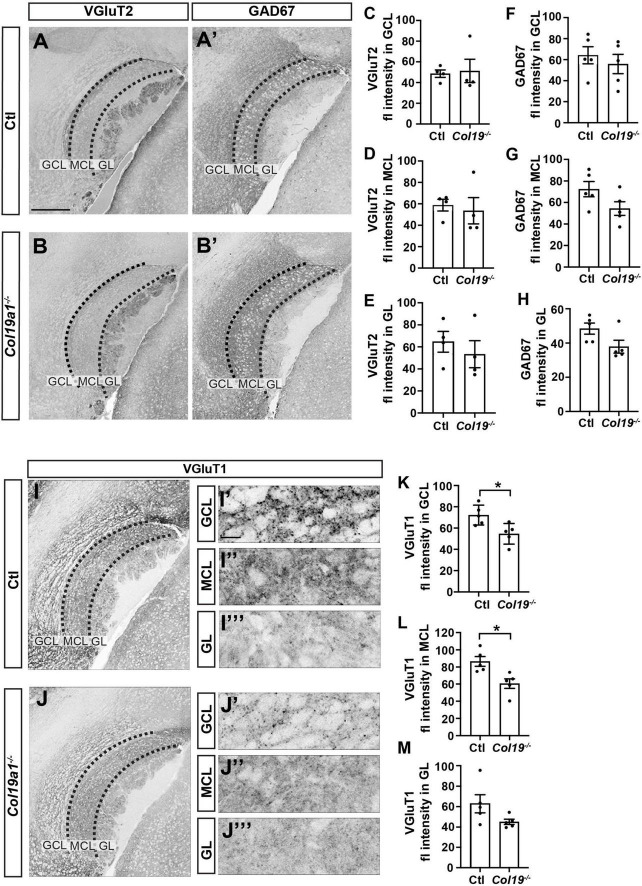
Excitatory synapses in the accessory olfactory bulb (AOB) are disrupted in *Col19a1*^– /–^ mice. **(A,B)** Sagittal brain tissue from control and *Col19a1*^– /–^ mice was stained with antibodies against VGluT2 and GAD67. **(C–H)** Quantification of VGluT2 and GAD67 immunoreactivity measured in the granule cell layer (GCL), mitral cell layer (MCL), and glomerular layer (GL) regions of the AOB. **(I,J)** Sagittal tissue from control and *Col19a1*^– /–^ mice was stained with antibody against VGluT1, with magnifications of the GCL, MCL, and GL of the AOB shown to the side, **(K–M)** quantifications of VGluT1 immunoreactivity in the MCL, GCL, and GL. Age P14. *Indicates *p* < 0.05 with unpaired Student’s *t*-test. Scalebars indicate 200 μm in panels **(A–J)** and 20 μm in the insets **(I’-I”’)** and **(J’-J”’)**. (*n* ≥ 3).

In order to uncover the mechanism by which Collagen XIX affects pheromone preference, we sought to test whether synapse formation was impaired in the AOB of these mutant mice, since previous studies showed that this collagen triggered inhibitory synapse formation in other brain regions (Su et al., 2010, 2016, 2020). To test this, we co-stained tissue sections from control and *Col19a1*^–/–^ mice with antibodies that label inhibitory synapses (i.e., GAD67) and a subset of excitatory synapses (i.e., VGluT2). The data showed that the intensity of GAD67 or VGluT2 immunolabeling was not significantly different between mutants and controls in each layer of the AOB (Figures 4A–H). We next labeled another subset of excitatory synapses with antibodies against VGluT1. Surprisingly, VGluT1 fluorescence intensity was significantly reduced in both the GCL and MCL of *Col19a1^–/–^* mutants. VGluT1-immunolabeling in other brain regions appeared unaffected by the loss of Collagen XIX, such as in the GL of the AOB (Figures 4I–M).

**FIGURE 5:**
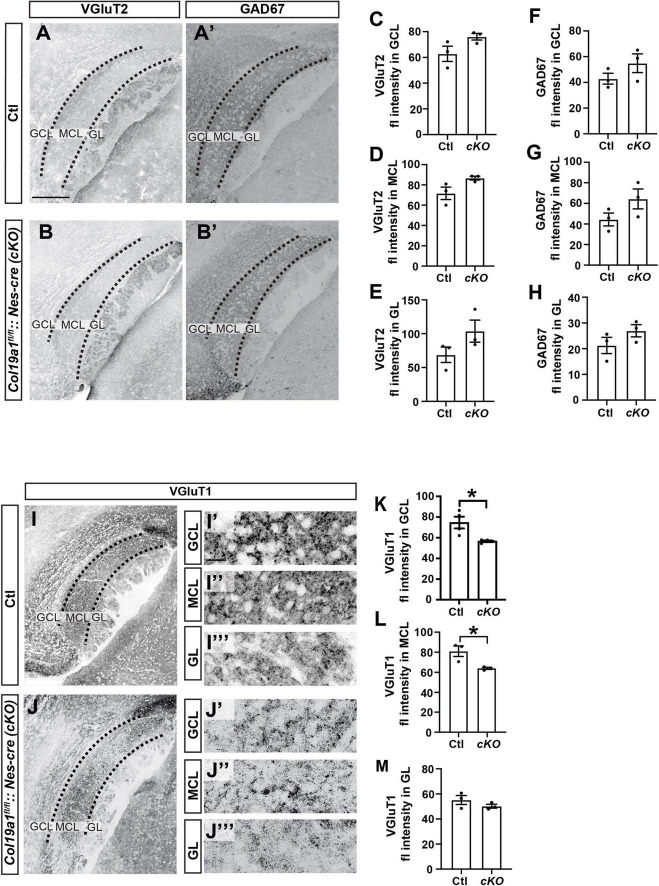
Excitatory synapses in the accessory olfactory bulb (AOB) are disrupted in conditional *Col19a1 mutants*. **(A,B)** Sagittal brains from control and *Col19a1*^fl/fl^*: Nestin-cre* (*Col19a1*^fl/fl^*: Nes-cre*) mice were stained with antibodies against VGluT2 and GAD67. **(C–H)** Quantification of VGluT2 and GAD67 immunoreactivity measured in the granule cell layer (GCL), mitral cell layer (MCL), and glomerular layer (GL) regions of the AOB. **(I,J)** Sagittal tissue from control and *Col19a1*^fl/fl^*:Nes-cre* mice stained with antibody against VGluT1, with magnifications of the GCL, MCL, and GL of the AOB shown to the side, **(K–M)**. Quantifications of VGluT1 immunoreactivity in the MCL, GCL, and GL. Age P14. *Indicates *p* < 0.05 with unpaired Student’s *t*-test. Scalebars indicate 200 μm in panels **(A–J)** and 20 μm in the insets **(I’-I”’)** and **(J’-J”’)**. (*n* ≥ 3).

We next tested whether neuronal Collagen XIX was specifically essential to excitatory synapse formation in the AOB. We immuno-stained *Col19a1^fl/fl^*::*Nes*-cre mouse tissues with antibodies against GAD67, VGluT2 and VGluT1. Similar to *Col19a1*^–/–^ mutants, loss of brain-derived Collagen XIX did not impact VGluT2 or GAD67 immunoreactivity in the AOB, but VGluT1 immunoreactivity was significantly decreased in MCL and GCL of the AOB (Figure 5). We observed synaptagmin 2 (Syt2) which mainly labels inhibitory synapses in mouse cortex. There was no significant difference between mutants and controls (Supplementary Figure 2).

When we immuno-stained *Col19a1^fl/fl^*::*Sst*-cre mouse tissues with antibodies against GAD67, Syt2, VGluT2, and VGluT1, we did not find significant changes in their immunoreactivity in AOB (Supplementary Figure 3). This further supports that pheromone recognition defects are the result of synaptogenesis defects in Collagen XIX global and neuronal deletion mice.

## Discussion

Collagen XIX has previously been shown to be both necessary and sufficient to trigger inhibitory synapse formation in the neocortex and hippocampus of mice (Su et al., 2010, 2016, 2020). Here, we added to our knowledge of this brain-derived ECM molecule by showing another specific population of neurons that generated *Col19a1* mRNA in the AOB. In contrast to cell-specific *Col19a1* expression by *Syt1*^+^ GABAergic neurons in mouse telencephalon, we found here that only 2/3 the *Col19a1*^+^ neurons in the AOB were GABAergic. By ablating the expression of *Col19a1* (either globally or in neural cells), we demonstrated its key role in the assembly of VGluT1^+^ excitatory presynaptic terminal machinery in the MCL and GCL of the AOB, the first description of a developmental function for *Col19a1* in excitatory synapse formation. This starkly contrasts Collagen XIX’s previously reported contribution to inhibitory synapse formation in the neocortex and hippocampus (Su et al., 2010, 2016, 2020), suggesting a regional specificity of Collagen XIX in synapse formation. Additionally, behavioral studies showed that Collagen XIX was necessary for male mice to have an olfactory preference for female scents (Figures 3C, D).

### Synaptogenic roles of Collagen XIX in the OB

Collagen XIX is part of a family of non-fibrillar collagen termed unconventional collagens. Many such collagens (and other ECM proteins) contain protein domains that can be enzymatically released to exert unique bio-activities, distinct from the function of the uncleaved matrix protein. These released fragments are termed matricryptins (Ramont et al., 2007; Su et al., 2016). Previous studies showed that Collagen XIX triggers inhibitory nerve assembly by releasing such a matricryptin, a short C-terminal fragment containing the non-collagenous domain 1 (NC1 domain) fragment of Collagen XIX, termed NC1[XIX] (Su et al., 2016). NC1[XIX] binds integrin receptors, leading to the assembly of presynaptic nerve terminals in a subset of GABAergic interneurons in telencephalon. As such, we interpreted the decrease in VGluT1 immunoreactivity in the *Col19a1*^–/–^ AOB shown here (Figures 4I–M) to suggest an impaired assembly of excitatory nerve terminals due to the loss of NC1[XIX] signaling.

In many cases, synaptogenic cues are target derived molecules, which means that they are produced from the postsynaptic neurons (Fox and Umemori, 2006), such as target derived adhesion molecules, growth factors, and matrix molecules (Scheiffele et al., 2000; Umemori et al., 2004; Fox et al., 2007; Terauchi et al., 2010; Su et al., 2012). We previously reported that Collagen XIX is not a target derived synaptogenic molecule in mouse neocortex (Su et al., 2016, 2020). Based on the results collected here, we think that Collagen XIX is likely not a target derived synaptogenetic molecule in mouse OB. This is due to the fact that *Col19a1* was specifically expressed in the MCL, but it impacts VGluT1^+^ terminals in both the MCL and GCL. It more likely acts in a paracrine fashion, similar to a number of glial-derived synaptogenic cues (Ullian et al., 2004; Christopherson et al., 2005; Kucukdereli et al., 2011).

### Loss of Collagen XIX impairs pheromone sensing

We found that wildtype adult male mice have a significant preference for female urine (Figure 3B). This preference for female urine was consistent with the well-characterized finding that male mice prefer investigating female pheromones and urine (Mandiyan et al., 2005; Pankevich et al., 2006; Martel and Baum, 2009; Zou et al., 2013). When Collagen XIX was removed globally (*Col19a1*^–/–^) or from all neural cells (*Col19a1^fl/fl^*:: *Nes*-cre), male mice no longer exhibited a preference for female urine (Figures 3C, D). We interpreted this to be due to its presence or function in the AOB, however, it could have been a secondary sequence of the loss of this collagen elsewhere in the brain. We found that *Sst*^+^ cells are not present in the AOB (Figure 2G) but are present in the anterior olfactory nucleus (Supplementary Figure 1) and cortex (Su et al., 2020), and are the main source of Collagen XIX in this region. Loss of Collagen XIX from *Sst*^+^ cells are sufficient to lead to impair GABAergic synapse formation and behavioral abnormalities in mice (Su et al., 2020). Thus *Col19a1^fl/fl^*::*Sst*-cre mice became a powerful control to address the regional specificity of *Col19a1* in pheromone function. In *Col19a1*^fl/fl^**::*Sst*-cre mice, the male preference for female urine was similar to wildtype (Figure 3E). We interpreted this to mean that telencephalic (or *Sst*+ cell) expression of *Col19a1* was not necessary for olfactory preference and that *Col19a1* expression in the AOB is likely essential to pheromone sensing.

The dependence of olfactory preference on the MCL in the AOB is consistent with previous findings in which ablation of the AOB led to impaired olfactory preference for female pheromones (Jakupovic et al., 2008). The AOB is a key part of the mouse limbic system (Maksimova et al., 2019) and responsible for directing input from sensory neurons in the VNO, which are responsible for sensing pheromones (Pankevich et al., 2004, 2006). The mitral cells in the AOB project to the medial amygdala (MeA) and the posteromedial cortical nucleus of the amygdala (PMCo) and subsequently to multiple regions of the hypothalamus in order to direct reproductive behavior (Kang et al., 2009; Martel and Baum, 2009; Maksimova et al., 2019). Other studies report that when the VNO was removed from mice, less granule cell and MCL activity (less Fos-immunoreactive cells) was observed in the AOB, and the olfactory preference of male mice for female urine was eliminated (Pankevich et al., 2004). The strong dependence of olfactory preference on MCL activity and connectivity supports that *Col19a1* is critical to the assembly and function of the VNO pathway (VNO to AOB to MeA to hypothalamus) in processing pheromones.

## Data availability statement

The original contributions presented in this study are included in the article/Supplementary material, further inquiries can be directed to the corresponding author.

## Ethics statement

The animal study was reviewed and approved by the Virginia Polytechnic Institute and State University Institutional Animal Care and Use Committee (IACUC).

## Author contributions

CA performed research and wrote the manuscript. MF acquired resources and funding and edited the manuscript. JS designed and performed research and wrote the manuscript. All authors contributed to the article and approved the submitted version.

## Supplementary material

Supplementary Figure 1 Most of *Col19a1* is expressed by Sst positive neurons in anterior olfactory nucleus. Images were acquired from sagittal tissue sections from P14 Sst-cre-tdT mice. Scalebars indicate 20 μm.

Supplementary Figure 2 Syt2 synapses are not disrupted in *Col19a1*^fl/fl^*: Nes-cre* conditional mutant AOB. **(A,B)** Sagittal brains from control and *Col19a1*^fl/fl^*: Nestin-cre* (*Col19a1*^fl/fl^*: Nes-cre*) mice were stained with antibodies against Syt2. **(C–E)** Quantification of Syt2 immunoreactivity measured in the GCL **(C)**, MCL **(D)**, and GL **(E)** regions of the AOB. Scalebars indicate 200 μm in panels **(A,B)**. (*n* = 3).

Supplementary Figure 3 Synaptogenesis of AOB region is not affected in *Col19a1*^fl/fl^*: Ssts-cre* mouse tissue. **(A,B)** Sagittal brain tissue from control and *Col19a1*^fl/fl^*: Sst-cre* mice was stained with antibodies against VGluT2 and GAD67. **(C–H)** Quantification of VGluT2 and GAD67 immunoreactivity measured in the GCL, MCL, and GL regions of the AOB. **(I,J)** Sagittal tissue from control and *Col19a1*^fl/fl^*: Sst-cre* mice was stained with antibody against VGluT1 and Syt2. **(K–P)** Quantification of VGluT1 and Syt2 immunoreactivity measured in the GCL, MCL, and GL regions of the AOB. Scalebars indicate 200 μm in panels **(A,B)** and **(I,J)**. (*n* = 3).
