# Supplementary figures and images for: Collagen XIX is required for pheromone recognition and glutamatergic synapse formation in mouse accessory olfactory bulb
Source: Front Cell Neurosci. 2023 Apr 5;17:1157577. doi: 10.3389/fncel.2023.1157577 (PMC10113670; doi:10.3389/fncel.2023.1157577)

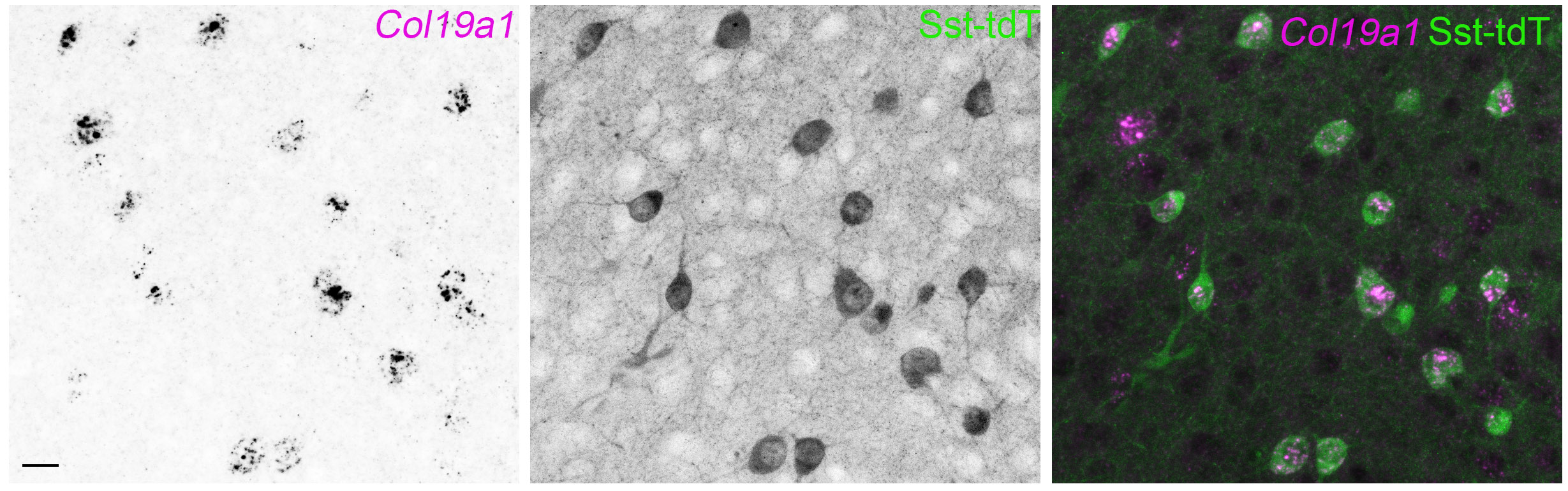

Supplement: Supplementary Figure 1 — Most of Col19a1 is expressed by Sst positive neurons in anterior olfactory nucleus. Images were acquired from sagittal tissue sections from P14 Sst-cre-tdT mice. Scalebars indicate 20 μm. [file Image_1.JPEG]

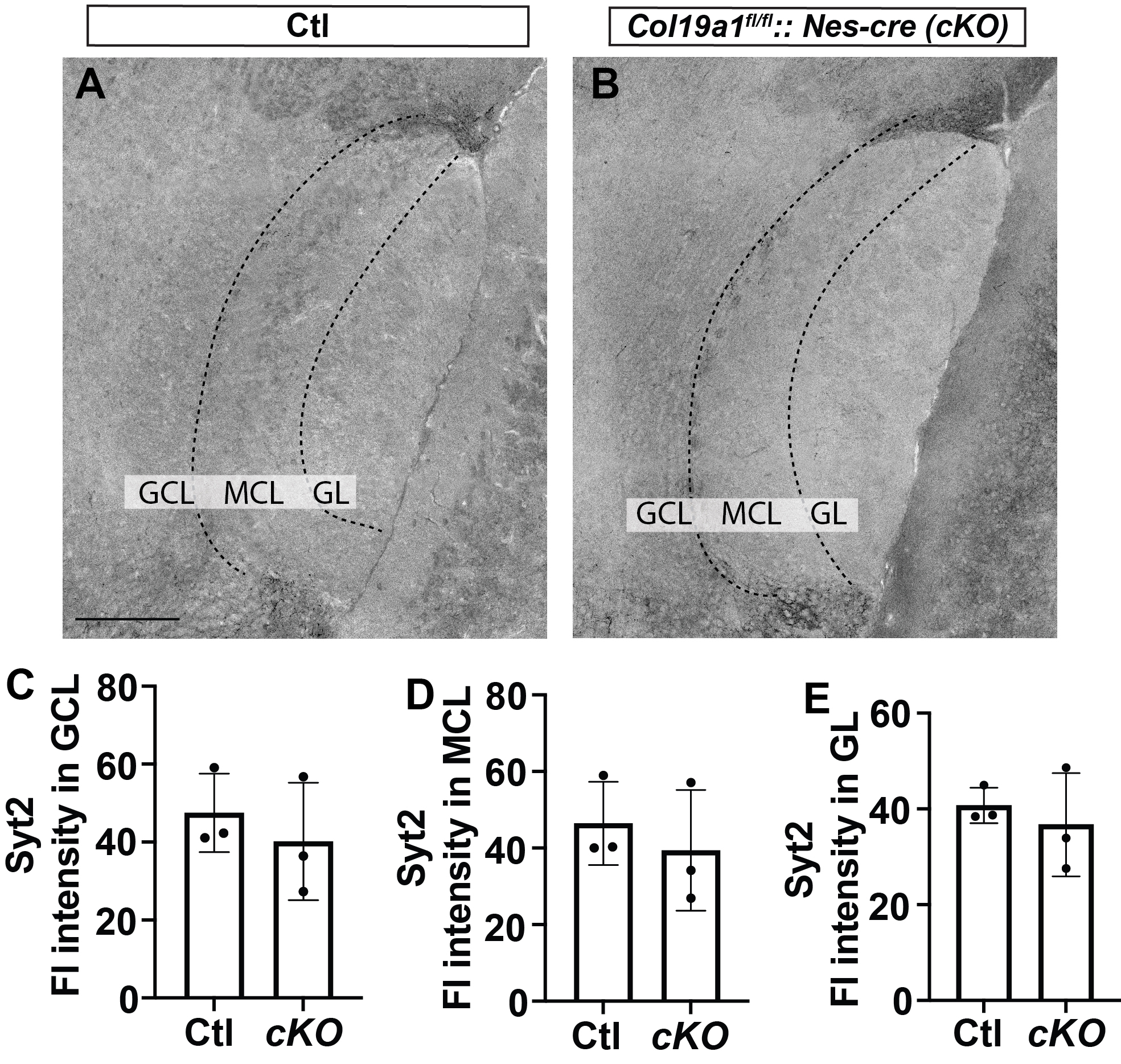

Supplement: Supplementary Figure 2 — Syt2 synapses are not disrupted in Col19a1fl/fl: Nes-cre conditional mutant AOB. (A,B) Sagittal brains from control and Col19a1fl/fl: Nestin-cre (Col19a1fl/fl: Nes-cre) mice were stained with antibodies against Syt2. (C–E) Quantification of Syt2 immunoreactivity measured in the GCL (C), MCL (D), and GL (E) regions of the AOB. Scalebars indicate 200 μm in panels (A,B). (n = 3). [file Image_2.JPEG]

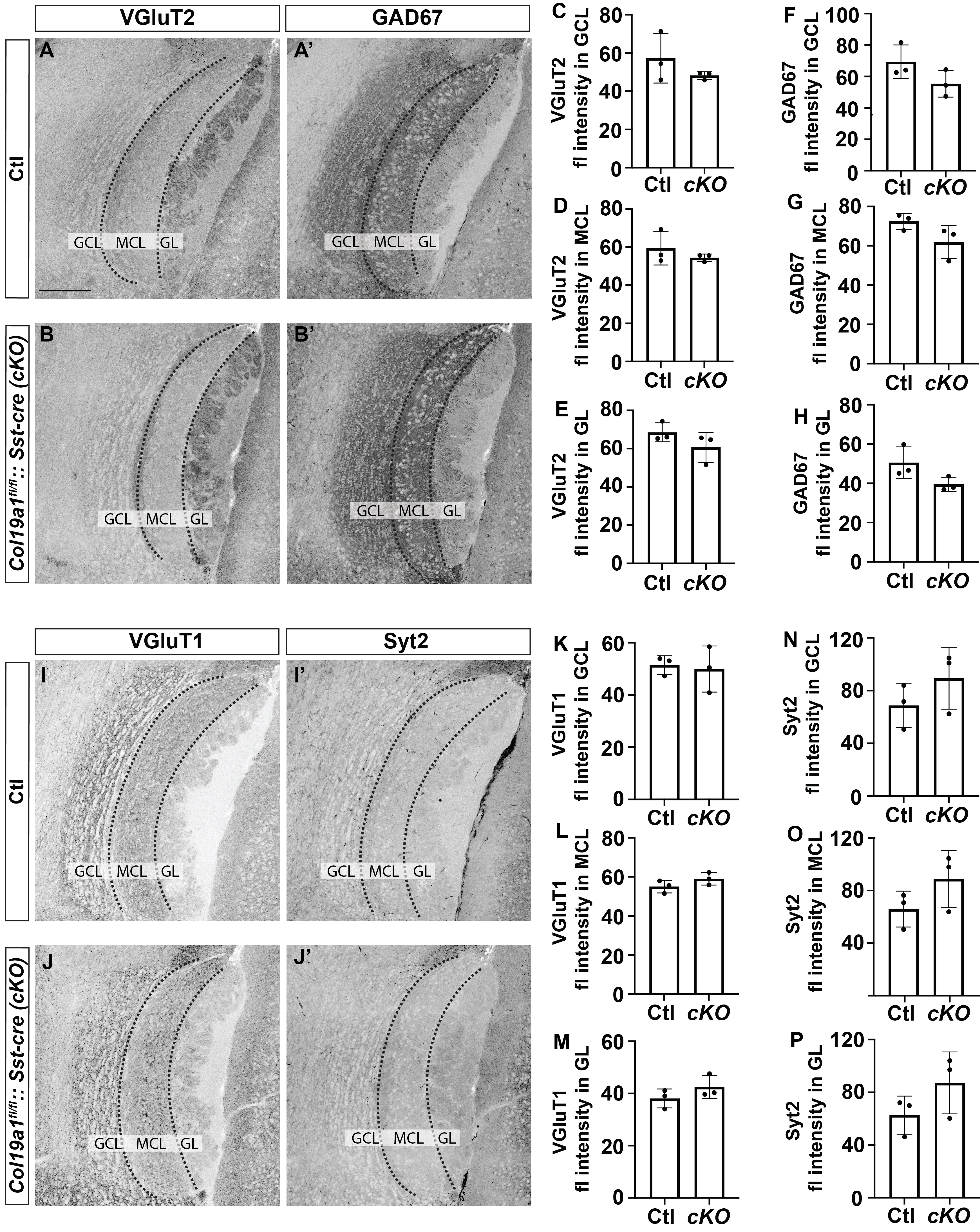

Supplement: Supplementary Figure 3 — Synaptogenesis of AOB region is not affected in Col19a1fl/fl: Ssts-cre mouse tissue. (A,B) Sagittal brain tissue from control and Col19a1fl/fl: Sst-cre mice was stained with antibodies against VGluT2 and GAD67. (C–H) Quantification of VGluT2 and GAD67 immunoreactivity measured in the GCL, MCL, and GL regions of the AOB. (I,J) Sagittal tissue from control and Col19a1fl/fl: Sst-cre mice was stained with antibody against VGluT1 and Syt2. (K–P) Quantification of VGluT1 and Syt2 immunoreactivity measured in the GCL, MCL, and GL regions of the AOB. Scalebars indicate 200 μm in panels (A,B) and (I,J). (n = 3). [file Image_3.JPEG]
